# *Boswellia sacra *essential oil induces tumor cell-specific apoptosis and suppresses tumor aggressiveness in cultured human breast cancer cells

**DOI:** 10.1186/1472-6882-11-129

**Published:** 2011-12-15

**Authors:** Mahmoud M Suhail, Weijuan Wu, Amy Cao, Fadee G Mondalek, Kar-Ming Fung, Pin-Tsen Shih, Yu-Ting Fang, Cole Woolley, Gary Young, Hsueh-Kung Lin

**Affiliations:** 1 Al Afia Medical Complex, Salalah, Sultanate of Oman; 2 Department of Urology, University of Oklahoma Health Sciences Center, Oklahoma City, OK 73104, USA; 3 Department of Physiology, University of Oklahoma Health Sciences Center, Oklahoma City, OK 73104, USA; 4 Department of Biological Sciences, University of Southern California, Los Angeles, CA 90089, USA; 5 Department of Pathology, University of Oklahoma Health Sciences Center, Oklahoma City, OK 73104, USA; 6 Department of Food Science, National Pingtung University of Science and Technology, Pingtung 91207, Taiwan, ROC; 7 Young Living Essential Oils, Lihi, UT 84043, USA

## Abstract

**Background:**

Gum resins obtained from trees of the Burseraceae family (*Boswellia sp*.) are important ingredients in incense and perfumes. Extracts prepared from *Boswellia sp*. gum resins have been shown to possess anti-inflammatory and anti-neoplastic effects. Essential oil prepared by distillation of the gum resin traditionally used for aromatic therapy has also been shown to have tumor cell-specific anti-proliferative and pro-apoptotic activities. The objective of this study was to optimize conditions for preparing *Boswellea sacra *essential oil with the highest biological activity in inducing tumor cell-specific cytotoxicity and suppressing aggressive tumor phenotypes in human breast cancer cells.

**Methods:**

*Boswellia sacra *essential oil was prepared from Omani Hougari grade resins through hydrodistillation at 78 or 100 ^o^C for 12 hours. Chemical compositions were identified by gas chromatography-mass spectrometry; and total boswellic acids contents were quantified by high-performance liquid chromatography. *Boswellia sacra *essential oil-mediated cell viability and death were studied in established human breast cancer cell lines (T47D, MCF7, MDA-MB-231) and an immortalized normal human breast cell line (MCF10-2A). Apoptosis was assayed by genomic DNA fragmentation. Anti-invasive and anti-multicellular tumor properties were evaluated by cellular network and spheroid formation models, respectively. Western blot analysis was performed to study *Boswellia sacra *essential oil-regulated proteins involved in apoptosis, signaling pathways, and cell cycle regulation.

**Results:**

More abundant high molecular weight compounds, including boswellic acids, were present in *Boswellia sacra *essential oil prepared at 100 ^o^C hydrodistillation. All three human breast cancer cell lines were sensitive to essential oil treatment with reduced cell viability and elevated cell death, whereas the immortalized normal human breast cell line was more resistant to essential oil treatment. *Boswellia sacra *essential oil hydrodistilled at 100 ^o^C was more potent than the essential oil prepared at 78 ^o^C in inducing cancer cell death, preventing the cellular network formation (MDA-MB-231) cells on Matrigel, causing the breakdown of multicellular tumor spheroids (T47D cells), and regulating molecules involved in apoptosis, signal transduction, and cell cycle progression.

**Conclusions:**

Similar to our previous observations in human bladder cancer cells, *Boswellia sacra *essential oil induces breast cancer cell-specific cytotoxicity. Suppression of cellular network formation and disruption of spheroid development of breast cancer cells by *Boswellia sacra *essential oil suggest that the essential oil may be effective for advanced breast cancer. Consistently, the essential oil represses signaling pathways and cell cycle regulators that have been proposed as therapeutic targets for breast cancer. Future pre-clinical and clinical studies are urgently needed to evaluate the safety and efficacy of *Boswellia sacra *essential oil as a therapeutic agent for treating breast cancer.

## Background

Frankincense is an aromatic resin hardened from exuded gums obtained from trees of the genus *Boswellia *(Burseraceae family). *Boswellia sp*. includes *Boswellia sacra *from Oman and Yemen, *Boswellia carteri *from Somalia, and *Boswellia serrata *from India and China. The resin has been used in incense and fumigants, as well as a fixative in perfumes. Aroma from these resins is valued for its superior qualities for religious rituals since the time of ancient Egyptians [1]. *Boswellia sp*. resins have also been considered throughout the ages to have a wealth of healing properties. For example, resins of *Boswellia sp*. have been used for the treatment of rheumatoid arthritis and other inflammatory diseases [2] such as Crohn's disease [3]. The anti-inflammatory activity has been attributed to the resin's ability in regulating immune cytokine production [4] and leukocyte infiltration [5,6]. Extracts from *Boswellia sp*. have been shown to possess anti-bacterial, anti-fungal [7], anti-carcinogenic [8], and anti-neoplastic [9,10] properties. Clinically, extracts from the resin have been shown to reduce the peritumoral edema in glioblastoma patients [9] and reverse multiple brain metastases in a breast cancer patient [11]. These results suggest that resins from *Boswellia sp*. contain active ingredients that modulate important biological and health supporting activities.

Boswellic acids have been identified as a major chemical component in *Boswellia sp*. extracts that provide the anti-inflammatory activity. Chevrier *et al*. reported that ethanol extracts of *Boswellia carteri *gum resins comprise 7 boswellic acids [4]. Akihisa *et al*. reported that methanol extracts of *Boswellia carteri *resins consist of 15 triterpene acids including boswellic acids [12]. Acetyl-11-keto-β-boswellic acid (AKBA), being suggested as the most potent anti-inflammatory component from the resins, selectively blocks leukotriene biosynthesis through inhibiting 5-lipoxygenase activity [13]. AKBA provides protective effects in a chemically induced mouse ulcerative colitis model [14]. Boswellic acids including AKBA have also been proposed to provide anti-neoploastic activity through their anti-proliferative and pro-apoptotic properties in multiple human cancer cell lines including meningioma cells [15], leukemia cells [16], hepatoma cells [17], melanoma cells, fibrosarcoma cells [18], colon cancer cells [19], and prostate cancer cells [20-22].

*Boswellia sp*. essential oil, an extract prepared by distillation of frankincense gum resins, is one of the most commonly used essential oils in aromatherapy. Considerable amount of work has been attempted to identify chemical compositions of *Boswellia sp*. essential oils from different commercial brands. Chemical constituents of *Boswellia sp*. essential oils differ significantly due to climates, time of harvest, storage conditions, geographical sources of resins [23], and methods of preparations. In this study, *Boswellia sacra *gum resins were collected in Oman; and essential oil was prepared *via *hydrodistillation at 78 or 100 ^o^C for 12 hours. Chemical profiles of these essential oils were analyzed. These essential oils were studied for their anti-tumor properties in a panel of human breast cancer cell lines and an immortalized normal breast epithelial cell line. *Boswellia sacra *essential oil-regulated Akt and ERK1/2 activation, cyclin D1 and cdk4 expression, and caspases activation were also assessed.

## Methods

### Reagents and chemicals

Cell culture media (RPMI 1640, MEM, Leibovitz's L-15, and DMEM/F-12), fetal bovine serum (FBS), horse serum, sodium pyruvate, MEM non-essential amino acids (NEAA), epidermal growth factor (EGF), cholera toxin, insulin, hydroxortisome, and penicillin-streptomycin were purchased from Invitrogen (Grand Island, NY). XTT cell proliferation assay and lactate dehydrogenase (LDH) cytotoxicity detection kits were obtained from Roche Applied Science (Indianapolis, IN). Matrigel™ basement membrane matrix was purchased from BD Biosciences (Bedford, MA). NanoCulture^® ^plate and media were obtained from SCIVAX Corp. (Kanagawa, Japan). Bicinchoninic acid (BCA) protein assay kit was purchased from Thermo Scientific Pierce (Rockford, IL). Rabbit anti-phospho-Akt (protein kinase B; PKB) (Ser473) antibody, rabbit anti-phospho-p44/42 MAP kinase (ERK1/2) (Thr202/Tyr204) antibody, mouse anti-cyclin D1 monoclonal antibody, mouse anti-cdk4 monoclonal antibody, mouse anti-human caspase-8 monoclonal antibody, rabbit anti-human caspase-9 polyclonal antibody, rabbit anti-cleaved caspase-3 (Asp175) monoclonal antibody, and rabbit anti-poly (ADT-ribose) polymerase (PARP) polyclonal antibody were purchased from Cell Signaling Technology (Danvers, MA). Mouse anti-human pro-caspase-3 monoclonal antibody was obtained from abcam (Cambridge, MA). Mouse anti-β-actin antibody was obtained from Sigma (St. Louis, MO).

### *Boswellia sacra *essential oil preparation

Hougari grade *Boswellia sacra *gum resins were obtained from the Hasik area to the east of Salalah City, Oman. The same batch of resins was equally divided into two portions and hydrodistilled at two temperatures, 78 or 100 ^o^C, at roughly atmospheric pressure in Salalah City. Briefly, hydrodistillation was performed in a custom made hydrodistiller. *Boswellia sacra *resins were loaded into 55 ^o^C water with a ratio of 1:2.5 (w/v), and mixed with an electromechanical agitator for 30-45 min or until a thick homogenous mucilage was formed. Temperatures of the hydrodistiller were monitored by an infrared thermometer; and pressures were recorded at the condenser terminal. To remove any residual water, collected *Boswellia sacra *essential oil was immediately transferred into a -20 ^o^C freezer; and ice crystals were separated from the essential oil.

### Chemical analysis of *Boswellia sacra *essential oil using gas chromatography-mass spectrometry (GC-MS)

Chemical components of the essential oils were analyzed with a 7890 GC-MS (Agilent Technologies, Santa Clara, CA) equipped with an HP-1 column (50 m × 0.32 mm × 0.5 um). GC oven temperature was set and maintained at 80 ^o^C for 2 min and programmed to 250 ^o^C at a rate of 3 ^o^C/min. The oven temperature was then programmed to 290 ^o^C at a rate of 10 ^o^C/min and maintained for 15 min. An aliquot (1 µl) of essential oil was injected at a 1:125 split ratio with injector and detector temperatures at 250 ^o^C with He as the carrier gas at 1.3 ml flow. MS was performed using a 5973 GC-MSD (Agilent Technologies) with an ionization voltage of 70 eV. Comparison of mass spectra for identification purposes used an essential oil database from CNRS (Lyon, France) as well as Wiley and NIST mass spectral libraries. Retention index calculations utilized C6 to C30 alkanes.

### Quantification of boswellic acids contents

Analysis of total boswellic acids contents in the essential oils was provided by San Rafael Chemical Services (Salt Lake City, UT). Briefly, a weighed portion of the sample was diluted in methanol, filtered, and then analyzed by high-performance liquid chromatography (HPLC) model 1090 II/L (Hewlett Packard) with synergi hydro-RP, 150 × 3.0 mm, 4 μm, 80 Å columns. Boswellic acids were detected by a photodiode array detector, scanning from 190 to 600 nm; and quantification was performed at 205 nm.

### Human breast cell lines

Human breast cancer T47D (HTB-133), MCF-7 (HTB-22), and MDA-MB-231 (HTB-26) cells as well as immortalized normal breast epithelial MCF-10-2A (CRL-10781) cells were purchased from ATCC (Manassas, VA). T47D cells were isolated from pleural effusion of a female patient with an infiltrating ductal carcinoma of the breast [24]. This cell line is estrogen receptor (ER) positive and cultured in RPMI 1640 plus 10% FBS and 1% sodium pyruvate. MCF-7 cells were derived from pleural effusion of breast adenocarcinoma from a female patient [25]. This cell line is ER-positive and cultured in MEM supplemented with 1% MEM NEAA, 1 mM sodium pyruvate, 10% FBS, and 10 ng/ml insulin. MDA-MB-231 cells were established from pleural effusion of a female patient diagnosed with adenocarcinoma [26], and maintained in Leibovitz's L-15 medium supplemented with 10% FBS. The immortalized normal MCF-10-2A breast cell line was derived from a patient with fibrocystic breast disease, and is non-tumorigenic in immunodeficient mice [27]. MCF-10-2A cells is maintained in DMEM/F-12 plus 5% horse serum, 20 ng/ml EGF, 100 ng/ml cholera toxin, 10 ng/ml insulin, and 500 ng/ml hydroxortisome. All culture media also supplemented with 100 units/ml penicillin-100 µg/ml streptomycin. Cells were cultured in a humidified cell incubator at 37 ^o^C and 5% CO_2 _and passaged every 3-4 days or when cells reached about 80% confluence.

### Cell growth and viability assay

Cell proliferation was determined in the breast cancer cell lines and immortalized MCF10-2A cells in their growth media. Cells (1x10^3^) were seeded into each well of 96-well tissue culture plates in 200 µl growth media; and viable cells were quantified between 1 and 4 days after seeding using the XTT cell proliferation assay kit. Briefly, 100 µl culture medium was removed from each well at the time of assay, and an aliquot of 50 µl XTT labeling mixture was added back to each well [28]. Reactions were performed at 37 ^o^C for 4 hours. Absorbance was read at 450 nm wavelength using a µQuant microplate reader (Bio-Tek; Winooski, VT). To determine *Boswellia sacra *essential oil-suppressed cell viability, the breast cell lines were seeded at 5x10^3 ^cells/well in 100 µl growth medium in 96-well tissue culture plates. Following overnight adherence, cells either received additional 100 µl growth media (untreated controls) or varying dilutions (1:200 to 1:2,700) of essential oil in growth media. Cell viability was determined using the XTT cell proliferation assay at 24 hours following essential oil exposure. Numbers of viable cells were calculated from standard curves with known numbers of cells run in parallel. Results were presented as average numbers of viable cells for cell growth and essential oil-suppressed cell viability.

### Cell cytotoxicity assay

*Boswellia sacra *essential oil-induced breast cell death was quantified by released LDH activity in culture media from damaged cells. Conditions for cell seeding and essential oil treatment were identical to the cell viability assay. At 3 hours following treatment, an aliquot of 100 µl medium was removed from each well and transferred to new 96-well plates. An aliquot of 100 µl LDH cytotoxicity detection reagent was added to each well and incubated for 15 min at room temperature. Absorbance was measured at 492 nm using the µQuant microplate reader. Numbers of damaged cells were calculated from standard curves established from known numbers of cells incubated with the cell lysis solution provided by the manufacture. Results were presented as average numbers of damaged cells for each essential oil preparation.

### Genomic DNA fragmentation

To determine *Boswellia sacra *essential oil-induced apoptosis in breast cancer cells, chromosomal DNA fragmentation, a biochemical hallmark of apoptosis, was performed. Breast cancer cells (3 × 10^5^) were seeded in 60 mm tissue culture plates in their growth media, incubated overnight for adherence, and treated with varying dilutions of essential oils obtained at 78 and 100 ^o^C hydrodistillation, respectively. Cells were harvested at 0 (untreated control), 2, 4, and 8 hours following treatment; and genomic DNA was isolated and precipitated based on reported procedures [29]. Total genomic DNA isolated from each sample was subjected to RNase A (10 μg/ml) digestion followed by size separation on 2% agarose gels. The gels were stained with 0.5 µg/ml ethidium bromide; and images of the stained gels were captured by the Gel Doc 100 system (Bio-Rad; Hercules, CA).

### Matrigel invasion assay

To determine the capability of *Boswellia sacra *essential oil in suppressing breast cancer cell invasion, a modified *in vitro *Matrigel outgrowth assay was utilized [30]. Briefly, an aliquot (100 µl) of liquefied Matrigel basement membrane matrix was transferred to each well of 96-well culture plates and allowed to polymerize at 37 ^o^C for 30 min. Breast cancer cells were trypsinized; and an aliquot of 2 × 10^4 ^or 4 × 10^4 ^cells was either resuspended in 100 µl growth medium alone or 100 µl growth medium containing varying dilutions of essential oils. After overnight incubation, cell spreading and invading into Matrigel matrix were imaged with a Nikon Eclipse TE 2000-S inverted microscope using a digital CCD camera (Roper Scientific, Sarasota, FL) equipped with NIS-Elements AR 3.0 imaging software. XTT assay was then performed in the same wells to determine the viability of essential oil-treated breast cancer cells on Matrigel.

### Spheroids formation assay

To evaluate and predict breast cancer cell responses to *Boswellia sacra *essential oil, multicellular tumor spheroids, an *in vitro *model for simulating three-dimensional tumor micro-milieu [31], were applied. Breast cancer cells (1 × 10^4^) were seeded into each well of the 96-well NanoCulture^® ^plates in 50 µl growth medium for spheroids formation. Cells were then either left untreated or treated with varying dilutions of essential oils at 48 hours post seeding. At 24 hours following essential oil treatment, images of multicellular spheroids were visualized and captured with the Nikon inverted microscope and imaging software.

### Western blot analysis

*Boswellia sacra *essential oil-regulated target proteins activation and expression were analyzed using Western blotting. Briefly, 5x10^5 ^breast cells were seeded in 60 mm tissue culture plates and exposed to either 1:800 or 1:1,200 dilutions of essential oils obtained at 78 or 100 ^o^C, respectively. Cells were then harvested at designated time points by lysing the cells with RIPA buffer supplemented with 0.1 mM phenylmethylsulphonylfluoride (PMSF), complete mini-protease inhibitor cocktail, and PhosSTOP phosphatase inhibitor Cocktail (Roche Applied Science). Following centrifugation, total cellular proteins were collected; and protein concentration was quantified by the BCA protein assay kit. An aliquot (30 µg) of the total cellular proteins from each sample was separated on 10% Tris-HCl gel (Bio-Rad); and the separated proteins were transferred to PVDF membranes (Bio-Rad). Expression of caspase-8, caspase-9, pro and cleaved caspase-3, PARP, phospho-Akt, phospho-ERK1/2, cyclin D1, cdk4, and β-actin was detected by incubating the PVDF membranes with primary antibodies against these molecules followed by appropriate peroxidase-conjugated secondary antibody incubation. Immunoreactive protein bands were detected using an enhanced chemiluminescent (ECL) reagent (Thermo Scientific Pierce). Images of immunoreactive protein bands were captured using the Gel Doc 100 system with Quantity One^® ^imaging software (Bio-Rad).

### Statistical analysis

The half maximal inhibitory concentrations (IC50) of *Boswellia sacra *essential oil were calculated from cell viability assay using the curve fitting function in Sigma Plot (Systac Software, San Jose, CA). Essential oil-induced cytostatic and cytotoxic effects were expressed as mean ± standard error of mean (SEM) from at least four experiments. Comparisons of breast cancer cell viability and death following essential oil treatment were made using the one-way analysis of variance (ANOVA) followed by Tukey multiple comparison test to compare cell lines' mean responses to essential oil treatment. Results were considered statistically significant when *P *< 0.05. **Results**

### Chemical composition of *Boswellia sacra *essential oil

Chemical profiles for *Boswellia sacra *essential oils obtained from different temperatures of hydrodistillation demonstrated that α-pinene is the major compound present in both temperatures preparations (Additional File 1, Table S1). Contents of α-pinene decreased with higher temperature distillation. In addition, essential oils from both temperatures preparations were primarily composed of the major monterpene, including α-thujene, unidentified 1, β-pinene, and myrcene. In general, all compounds with higher retention indices, with a few exceptions, were present in higher quantities in essential oil distillated at 100 ^o^C as compared to that obtained at 78 ^o^C.

### Quantitatiion of boswellic acids

Since triterpene including boswellic acids could not be detected by the current GC-MS protocol used in our laboratory, an HPLC method was applied to determine total boswellic acids in *Boswellia sacra *essential oils. Contents of boswellic acids in essential oils depended upon temperature of hydrodistillation. Higher distillation temperature produced higher quantities of boswellic acids; higher amounts of total boswellic acids were detected in essential oil hydrodistilled at 100 ^o^C as compared to 78 ^o^C (Table 1).

**Table 1:** Boswellic acids contents in *Boswellia sacra *essential oils

	Specific gravity	Total boswellic acids (mg/ml)
78 ^o^C distillation	0.852	19.6
100 ^o^C distillation	0.847	30.1

### Suppression of tumor cell-specific viability by *Boswellia sacra *essential oils

**Figure 1:**
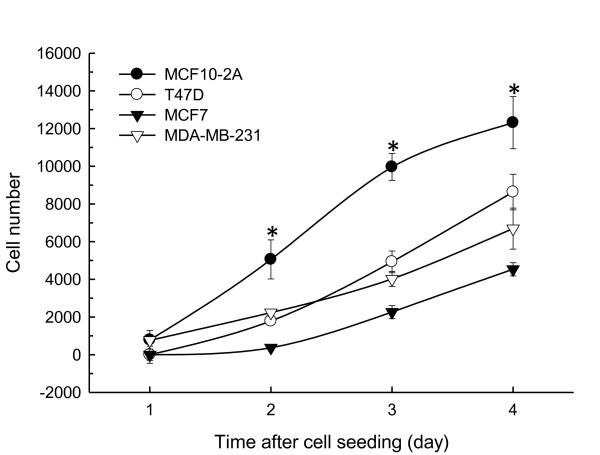
**Human breast cell growth**. Human breast cancer cells and immortalized normal breast epithelial cells (1x10^3^) were seeded in triplicate in their culture media, and quantified for their growth between 1 and 4 days following cell seeding. Cell numbers were calculated from standard curves with known numbers of cells. Results are presented as mean ± SEM from 4 independent experiments. * indicates statistical differences between normal and malignant cells at *P *< 0.05.

Cell proliferation was determined in all four human breast cell lines in the absence of *Boswellia sacra *essential oil. Under the culture condition used for each cell line, the immortalized normal breast epithelial cells proliferated faster than three breast cancer cell lines. Doubling time for MCF10-2A cells was 12 hours as compared to 20, 22, and 27 hours for T47D, MCF7, and MDA-MB-231 cells, respectively; and MCF10-2A cell numbers were significantly higher than T47D, MCF7, or MDA-MB-231 cells at days 2, 3, and 4 following cell seeding (Figure 1).

**Figure 2:**
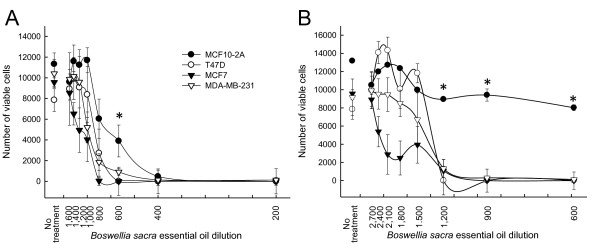
**Breast cell viability in response to *Boswellia sacra *essential oil exposure**. Human breast cells (5x10^3^) were seeded in each well of 96-well tissue culture plates in triplicate. Following adherence, cells were treated with *Boswellia sacra *essential oil hydrodistilled at (A) 78 ^o^C or (B) 100 ^o^C for 12 hours. Cell viability was determined using the colometric XTT assay at 24 hours after essential oil treatment. Data are presented as mean ± SEM from at least 4 independent experiments. * indicates statistical differences between essential oil-treated breast cancer cells and immortalized breast cells (*P *< 0.05).

*Boswellia sacra *essential oils were studied for their capabilities in suppressing breast cancer cell viability in cultures. For essential oil collected at 78 ^o^C, 200 to 1:1,600 dilutions were used, whereas a wider range of dilutions (600 to 2,700) was used for essential oil collected at 100 ^o^C. Although different cancer cell lines varied in their sensitivities to essential oil treatment, both temperatures of essential oil preparations, in general, suppressed cell viability in all three human breast cancer cell lines (Figure 2). *Boswellia sacra *essential oil-suppressed cancer cell viability depended upon hydrodistillation temperatures. Essential oil obtained at 78 ^o^C possessed less potent anti-proliferative activity (Figure 2A) as compared to that prepared at 100 ^o^C (Figure 2B). More importantly, immortalized normal breast epithelial MCF10-2A cells were resistant to *Boswellia sacra *essential oil-suppressed cell viability (Figure 2). MCF10-2A cell viability was significantly higher than three breast cancer cell lines when 600 to 1,200 dilutions of essential oil (obtained at 100 ^o^C) was administered; in contrast, results were less significant when essential oil prepared at 78 ^o^C was used.

IC50 values were calculated to provide a quantitative assess of these essential oils. Results supported that essential oil hydrodistilled at 100 ^o^C produced more potent cytotoxic effects. For example, IC50 values for T47D cells were 900 and 1,450 dilutions for essential oils obtained at 78 and 100 ^o^C, respectively (Table 2). Among the cancer cell lines, MCF7 cells were the most sensitive to essential oil with suppressed cell viability.

**Table 2:** IC50 values of *Boswellia sacra *essential oils on human breast cells

Breast cell line	Temperature of	hydrodistillation
	
	78°C	100°C
MCF10-2A	1:680*	NA**
T47D	1:900	1:1,450
MCF7	1:1,000	1:1,800
MDA-MD-231	1:950	1:1,300

*numbers represent dilutions of essential oil

**NA: value is not available

### *Boswellia sacra *essential oil-induced breast tumor cell-specific death

**Figure 3:**
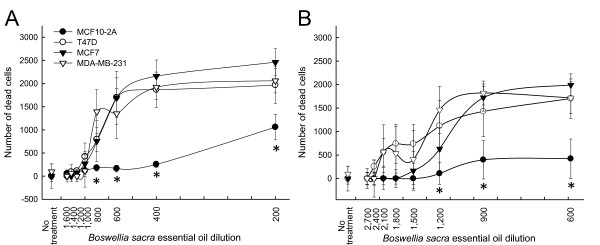
**Quantitative analysis of *Boswellia sacra *essential oil-induced human breast cancer cell death**. Human breast cell lines were seeded in each well of 96-well tissue culture plates at the concentration of 5x10^3 ^cells/well in triplicate. Following overnight adherence, cells were treated with either *Boswellia sacra *essential oil hydrodistilled at (A) 78 ^o^C or (B) 100 ^o^C. Cell death was determined at 3 hours following essential oil exposure by the LDH cytotoxicity detection kit. Data were presented as mean average numbers of dead cells ± SEM from at least 3 independent experiments. * indicates statistical differences of cell death between cancer cells and immortalized breast cells (*P *< 0.05).

In order to determine whether the reduced cell viability resulted from increased cell death and how cells responded at an early phase of treatment, essential oil-induced breast cell death was quantified by LDH release. Elevated cell death was observed in all three breast cancer cell lines treated with *Boswellia *sacra essential oils (Figure 3). In contrast, essential oil-induced cytotoxicity was significantly lower in immortalized MCF10-2A cells at 3 hours after treatment. Consistent to results from cell viability assays, *Boswellia sacra *essential oil prepared from 78 ^o^C hydrodistillation (Figure 3A) was less potent than the essential oil obtained at 100 ^o^C (Figure 3B) for inducing an early phase of tumor cell-specific death.

### *Boswellia sacra *essential oil-induced apoptosis

**Figure 4:**
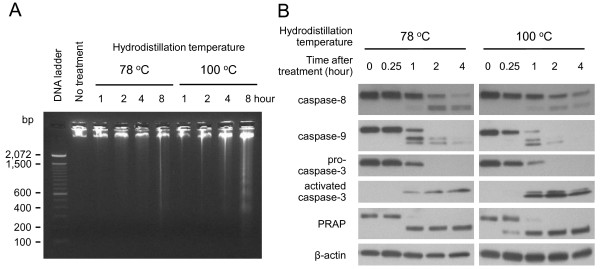
***Boswellia sacra *essential oil-induced apoptosis in human breast cancer cells**. Human breast cancer MDA-MB-231 cells were seeded at the concentration of 5x10^5 ^cells/60 mm tissue culture plates for adherence and subjected essential oils (1:800 and 1:1,200 dilutions of 78 and 100 ^o^C, respectively) treatment. Both genomic DNA and total cellular proteins were isolated at specific time points as indicated. (A) Essential oil-induced genomic DNA fragmentation. (B) Essential oil-activated pro- and cleavage of caspases. Experiments were repeated at least twice and representative results are presented.

Fragmentation of genomic DNA demonstrated that *Boswellia *sacra essential oil induced apoptosis in breast cancer cells. Essential oils prepared at 78 ^o^C (at 800 dilution) and 100 ^o^C (at 1,200 dilution) induced genomic DNA fragmentation in a time-dependent manner; all three human breast cancer cell lines exhibited similar patterns and visible fragmented genomic DNA within 8 hour post-treatment (Figure 4A). In contrast, the same concentrations of essential oils treatment did not induce DNA fragmentation in MCF10-2A cells.

Caspases, a family of cysteine proteases, play critical roles in apoptosis. Cleaved caspase-8 p43/p41 and caspase-9 p37/p35 were detected within 1 hour in essential oil treated MDA-MB-231 cells (Figure 4B). Caspase-3 is a key enzyme either partially or totally responsible for the proteolytic cleavage of several target proteins involved in executing apoptotic processes. Essential oil-induced activated (cleaved) caspase-3 expression showed prominent elevation of this protein corresponding to decreases of pro-caspase-3 levels within 1 hour post-stimulation by essential oils from both temperatures in MDA-MB-231 cells. Cleavage of PARP, involved in DNA repair following environmental stress [32] and a main cleavage target of caspase-3 [33], was also detected in MDA-MB-231 cells within 1 hour and 15 min following treatment with essential oils obtained at 78 and 100 ^o^C, respectively. In contrast, the same concentrations of *Boswellia sacra *essential oils did not induce detectible cleavage of caspase-8, caspase-9, caspase-3, or PARP in T47D, MCF7, or MCF10-2A cells.

### Anti-invasive activity of *Boswellia sacra *essential oil on Matrigel

**Figure 5:**
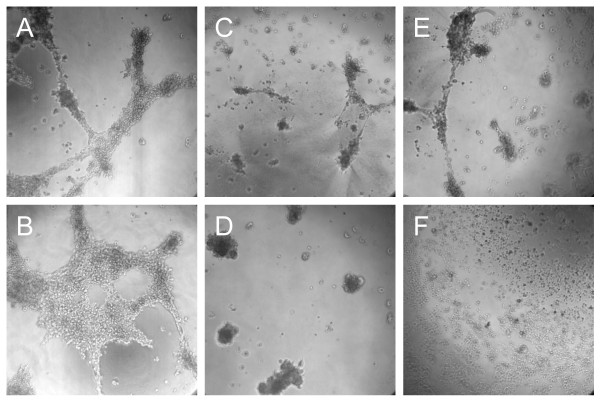
**Assessment of anti-invasive activity of *Boswellia sacra *essential oil**. Matrigel basement membrane matrix (100 μl) was transferred to each well of 96-well culture plates and allowed to polymerize at 37 ^o^C. MDA-MB-231 cells (4 × 10^4^) were resuspended in 100 µl growth media and added on the top of the polymerized Matrigel (A and B). In separate wells, cells were either mixed with 100 µl of 78 ^o^C essential oil at (C) 1:800 or (D) 1:600 dilution, or 100 ^o^C essential oil at (E) 1:1,500 or (F) 1:1,200 dilution and layered on the top of Matrigel. Formation of vascular-like networks was assessed at 24 hours following oil treatment. The capabilities of MDA-MB-231 cells in forming networks of tubes and *Boswellia sacra *essential oil in suppressing the cellular network formation on Matrigel are observed at 100× magnification. Experiments were repeated at least 3 times and representative images are presented.

MDA-MB-231 cells were able to form networks of tubes on Matrigel (Figure 5A and 5B). When cells were treated with essential oils at 1:800 (Figure 5C) and 1:1,500 (Figure 5E) dilutions obtained at 78 and 100 ^o^C hydrodistillation, respectively, the formation of cellular networks was reduced without inducing cytotoxicity. *Boswellia sacra *essential oils completely blocked MDA-MB-231 cell tube formation when 1:600 dilution of essential oil obtained at 78 ^o^C (Figure 5D) and 1:1,200 dilution of oil prepared at 100 ^o^C (Figure 5F) were applied, while cells remained viable on Matrigel based on the XTT assay. In contrast, higher concentrations of both essential oils (1:400 and 1:900 dilutions from 78 and 100 ^o^C hydrodistillation, respectively) suppressed both tube formation and viability of MDA-MB-231 cells on Matrigel. In contrast, MCF10-2A cells did not form capillary-like networks on Matrigel.

### *Boswellia sacra *essential oil-suppressed multicellular tumor spheroids growth

**Figure 6:**
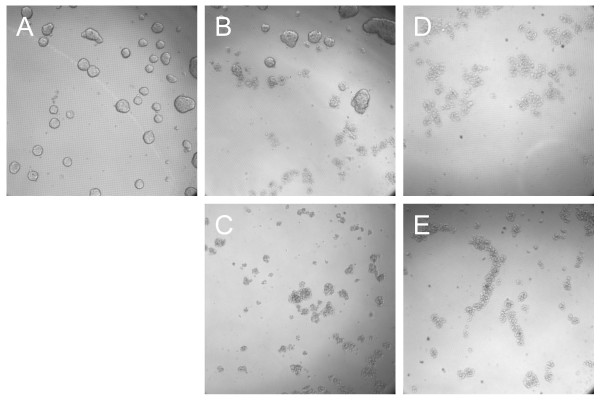
**Capability of *Boswellia sacra *essential oil in suppressing multicellular spheroid growth**. Breast cancer T47 cells (1 × 10^4^) were seeded into each well of the 96-well NanoCulture^® ^plates. Following the formation of spheroids, cells were either (A) left untreated or treated with 78 ^o^C essential oil at (B) 1:800 or (C) 1:600 dilution, or 100 ^o^C essential oil at (D) 1:1,500 or (E) 1:1,200 dilution. Spheroids images were captured at 24 hours following essential oil treatment at 100x magnification. Experiments were repeated at least 3 times and representative images are presented.

Essential oil was assessed for its capability in inducing tumor cell death in three-dimensional multicellular spheroids. Among the three breast cancer cell lines tested, T47D was the only cell line that formed spheroids on the NanoCluture^® ^plates (Figure 6A). Treatment of T47D cells with 1:800 dilution of essential oil obtained at 78 ^o^C (Figure 6C) as well as 1:1,500 and 1:1,200 dilutions obtained at 100 ^o^C (Figure 6D and 6E) blocked spheroids formation.

### *Boswellia sacra *essential oil-regulated expression of signaling molecules and cell cycle regulators

**Figure 7:**
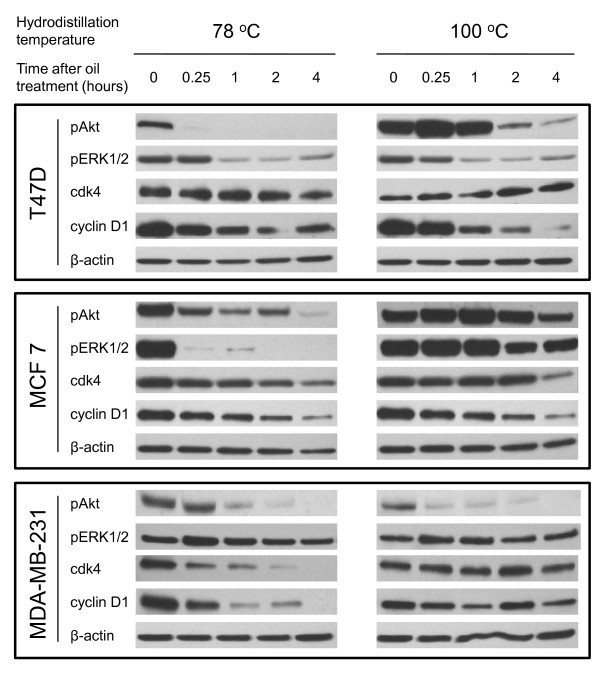
***Boswellia sacra *essential oil-regulated signaling molecules activation and cell cycle-related proteins expression in human breast cancer cells**. Breast cancer cells were seeded at the concentration of 5 × 10^5 ^cells/60 mm tissue culture plate. After adherence, cells were treated with either 1:800 dilution of 78 ^o^C or 1:1,200 dilution of 100 ^o^C essential oil. Total cellular proteins were isolated between 0 (untreated control) and 4 hours following essential oils treatment. Western blot analysis was performed to determine levels of Akt and ERK1/2 phosphorylation as well as cyclin D1 and cdk4 proteins expression. Expression of β-actin was also determined in parallel and used as a protein loading control. Experiments were repeated at least twice for each cell line and representative results are presented.

Essential oil-regulated Akt and ERK1/2 activation was analyzed to determine the impacts of these oils on these two important signaling pathways. Levels of phospho-Akt (Ser473) were immediately suppressed by essential oil obtained at 78 ^o^C in all breast cancer cell lines. In contrast, T47D and MCF7 cells responded with elevated levels of phospho-Akt (Ser473) expression within 15 min and gradually decreased thereafter when cells were treated with essential oil obtained at 100 ^o^C (Figure 7). Essential oils suppressed phosphorylated levels of ERK1/2 (Thr202/Tyr204) expression in T47D and MCF7 cells, whereas MDA-MB-231 cells responded with immediate up-regulation within 15 min and returned to untreated levels thereafter. In contrast, MCF10-2A cells did not have detectible phosporylated ERK1/2 expression, and their phosphorylated Akt expression was not altered by essential oil treatment.

*Boswellia sacra *essential oil also regulated the expression of cell cycle regulators, cdk4 and cyclin D1. Levels of cdk4 expression were gradually suppressed by essential oil obtained at 78 ^o^C in all 3 breast cancer cell lines, whereas cdk4 expression maintained relatively constant when T47D and MDA-MB-231cells were treated with oil distilled at 100 ^o^C (Figure 7). Cyclin D1 is a crucial cell cycle regulator in human breast cancer cells. All three breast cancer cell lines responded to essential oils with suppressed cyclin D1 expression. MCF10-2A cells did not respond to essential oil treatment with altered cdk4 expression, and had no detectible cyclin D1 expression.**Discussion**

The genus *Boswellia *consists of 18 genera and 540 species that grow mostly in tropical regions of America, China, India, North Africa, and Arabia. Each species of *Boswellia *produces a slightly different type of resin; and differences in soil and climate create more diversity in the resins, even within the same species. Although there are many commercial brands of essential oils from different species of *Boswellia *for aromatherapy practices, conditions for manufacturing these commercial essential oils are not consistent or standardized. We previously reported that *Boswellia cateri *essential oil possesses human bladder cancer cell-specific anti-proliferative and pro-apoptotic activities. In this communication, essential oil was prepared by hydrodistillating Omani *Boswellia sacra *resins at two temperatures and characterized their effectiveness in suppressing breast cancer cell-specific viability, apoptosis, invasion, drug resistance, and related signaling pathways *in vitro*.

Chemical composition of *Boswellia sacra *essential oils obtained from 78 and 100 ^o^C hydrodistillation were quantitatively and quantitatively characterized using GC-MS and HPLC. In general, *Boswellia sacra *essential oil obtained at 100 ^o^C hydrodistillation contains higher quantities of chemical compounds with retention time longer than sabinene as compared to essential oil prepared at 78 ^o^C. Boswellic acids from *Boswellia sp*. resins have been suggested to be a major compound in mediating various biological functions including anti-inflammatory and anti-cancer activities. It has been shown that β-boswellic acids from methanol extracts of *Boswellia cateri *gum resins exhibit potent cytotoxic activities against human neuroblastoma cell lines, IMR-32, NB-39, and SK-N-SH [12]. Shao *et al*. compared 4 triterpene acids including β-boswellic acid, 3-O-acetyl-β-boswellic acid, 11-keto-β-boswellic acid, and AKBA isolated from *Boswellia serrata *gum resins for their ant-cancer activity *in vitro*. AKBA is the most pronounced inhibitory effects among the 4 triterpene acids in suppressing human leukemia HL-60 cell growth as well as DNA, RNA, and protein synthesis [16]. AKBA also exhibits anti-proliferative and pro-apoptotic activities against human prostate cancer LNCaP and PC-3 cells *in vitro *and in animal models [21,34], and induces cytotoxicity in human meningioma cells in culture [15]. Higher boswellic acids contents are present in essential oil hydrodistilled at higher temperature. However, our results suggest that high molecular weight compounds other than boswellic acids may play significant roles in suppressing tumor cell viability and invasion. First, although shelf life contents of boswellic acids decreased, *Boswellia sacra *essential oil-mediated tumor cell cytotoxicity remained constant during the same period of time. Second, hydrosol, the aqueous phase of hydrodistilled products, contained up to 15.5% boswellic acids, but did not have detectible cytoxicity against breast cancer cells even when a 1:5 dilution was included in the cell cultures. Our results are in accordance with the report by Hostanska *et al*. that components other than AKBA from solvent extracts of *Boswellia serrata *gum resins can induce cytotoxicity in malignant cells [10]. Additionally, Estrada *et al*. reported that tirucallic acids purified from *Boswellia carteri *gum resins induce apoptosis in human prostate cancer cell lines [35]. Although the active compound(s) in *Boswellia sacra *essential oil responsible for anti-tumor activity cannot be identified immediately due the complexity of essential oils, chemical compositions and/or ratios of these components present in the oil obtained at 100 ^o^C would play significant roles in tumor cell-specific cytotoxicity.

Commonly, cancer chemotherapy drugs, including alkylating antineoplastic agent [36], antimetabolite [37], and anthracycline [38], act by impairing cell viability in rapidly dividing cells. Under the cell culture conditions used in this report, immortalized normal breast epithelial MCF10-2A cells proliferate significantly faster than breast cancer T47D, MCF7, or MDA-MB-231 cells. However, all cancer cell lines are more sensitive to *Boswellia sacra *essential oil treatment as compared to the immortalized normal cells. Consistently, human bladder cancer cells [39] and colonic cancer cells (data not shown) are more sensitive to *Boswellia sp*. essential oil with elevated cytotoxicity and apoptosis as compared to their normal counterparts. *Boswellia sp*. essential oil may possess certain unique components that specifically target and induce programmed cell death in malignant cells. The absence of activated caspase-3 expression in T47D and MCF7 cells suggests that essential oil-induced apoptosis may activate a caspase-3-independent pathway similar to taxol-induced apoptosis in breast carcinoma MCF7 cells [40].

*Boswellia sacra *essential oil also suppresses important malignant features of tumor cells, such as invasion and multicellular tumor spheroids growth. Tumor cell plasticity enables highly malignant tumor cells to express endothelial cell-specific markers and form vessel-like network structures on basement membranes. The *in vitro *Matrigel-based tumor invasion model has been shown to correlate with *in vivo *metastatic potential [41]. This *in vitro *model has been used to study mechanisms of cancer aggressive behavior, metastasis, and poor prognosis [42], and has been used as a tool to screen therapeutic agents for their anti-metastatic property [30,43]. MDA-MB-231 cells grown on Matrigel are more resistant to essential oil-suppressed cell viability as compared to cells grown on tissue culture plates. These differences may result from protective effects of the Matrigel basement membrane matrix enriched with various growth factors. In addition, cancer cells can form multicellular spheroid aggregates, which afford protection of cancer cells against some chemotherapeutic agents [44]. Multicellular tumor spheroids in culture have been used as an *in vitro *model for screening and testing anti-cancer drugs [45]. Similar to results from cytotoxicity and apoptosis, *Boswellia sacra *essential oil obtained at 100 ^o^C is more potent than essential oil obtained at 78 ^o^C hydrodistillation in disruption cellular networks on Matrigel and spheroids. More importantly, observations obtained in the above described experimental models are consistent with clinical responses in human cancer cases; and clinical case studies will be reported separately. These results suggest that *Boswellia sacra *essential oil may represent an effective therapeutic agent for treating invasive breast cancer.

Aberrant activations of Akt and ERK1/2 MAPK signaling molecules have been identified in various cancers including breast cancer; and activations of Akt and ERK1/2 have been suggested as independent cancer prognostic markers. The Akt pathway is found to be activated in early stages of breast cancer development [46]; and activation of Akt signaling protects breast cancer cells from tamoxifen-induced apoptosis *in vitro *and confers poor prognosis in cancer patients [47,48]. Activation of ERK1/2 is also shown to be associated with the development of tamoxifen resistant and patient survival [49,50]. Both Akt and ERK1/2 have been proposed as molecular targets for treating breast cancer particularly in antiestrogen-resistant states [51,52]. Targeting Akt signaling by inhibiting mTRO signaling has been shown to restore cancer responses to chemotherapy drugs [53,54]; and inhibition of both epidermal growth factor receptor (EGFR)/HER2 and MAPK signaling has been shown to result in growth inhibition and apoptosis of EGFR-expressing breast cancer cells [55]. Studies have shown that boswellic acids and AKBA activate the PI3K/Akt pathway in human colon cancer HT29 cells [56]. Although AKBA was reported to rapidly and potently inhibit the phosphorylation of ERK1/2 in primary cultures of meningioma cells [15], other studies showed that boswellic acids and AKBA activate ERK1/2 in human polymorphonuclear leukocytes and platelets [57,58]. Our results demonstrate that *Boswellia sacra *essential oil suppresses Akt and ERK1/2 activation in human breast cancer cell lines except MDA-MB-231 cells. The differences between boswellic acids and *Boswellia sacra *essential oil may result from different tumor cell types or components other than boswellic acids in the essential oil.

The majority of human mammary carcinomas overexpress cyclin D1 protein [59,60]; and overexpression of cyclin D1 has been shown to be correlated to breast cancer development and progression, including metastatic lesions [61,62]. Among many different cyclin D1 interactors, the *cdk4 *gene is found to be amplified and the protein to be overexpressed in a significant fraction of human breast cancer cases [63-65]; and the continued presence of cdk4-associated kinase activity is required to maintain breast tumorigenesis [66]. Therefore, cyclin D1-cdk4 has been proposed as a target for therapeutic intervention in mammary carcinomas. A highly specific inhibitor of cdk4/6 activity (PD-0332991) has been developed and evaluated for its efficacy in the treatment of breast cancer [67]. Boswellic acids, including AKBA, have been shown to arrest cancer cells at the G1 phase of cell cycle, suppresses cyclin D1 and E, cdk 2 and 4, as well as phosphorylated Rb and increased p21 expression through a p53-independent pathway [22,68,69]. Consistently, our results demonstrate that *Boswellia sacra *essential oil suppresses cyclin D1 and cdk4 expression in almost all breast cancer cell lines examined. Biological significances between essential oil-regulated PI3K/Akt and ERK1/2 activation, cdk4 and cyclin D1 expression, tumor cell cytotoxicity require further studies.

*Boswellia sacra *essential oil-activated cell death pathways are still under intensive investigation. Pathways that are activated by a mixture of chemical components in essential oil are expected to more complicate than the results presented in this communication. In our previous report, using *Boswellia cateri *essential oil, multiple genes and pathways that are associated with suppression of cell proliferation and cell cycle progression, as well as increase of apoptosis in human bladder cancer J82 cells were characterized [39]. Using a comprehensive gene expression analysis in J82 cells treated with *Boswellia cateri *essential oil, bioinformatics results suggest that the Nrf2-mediated oxidative stress pathway appears is the most plausible cause for selective cancer cell death (data not shown). *Boswellia sp*. essential oil may selectively eradicate cancer cells *via *suppressing intracellular accumulation reactive oxygen radicals as reported in other models [70,71].

## Conclusion

We have demonstrated that *Boswellia sacra *essential oil prepared from hydrodistillation has tumor cell-specific cytotoxicity in multiple cancer cell types. Consistent with anti-proliferative, pro-apoptotic, and anti-invasive activities in cultured breast cancer cells, *Boswellia sacra *essential oil is shown to induce tumor cell cytotoxicity in a drug resistant and metastasized breast cancer case. In addition to establishing standard procedures to produce essential oil with consistent chemical composition, safety and toxicity studies of the oil and pre-clinical validation of the *in vitro *results will be required. Moreover, formulation and standardization of clinical protocols to monitor cancer progression following *Boswellia sacra *essential oil administration are needed immediately for prospective human clinical trials.

## Abbreviations

AKBA: acetyl-11-keto-β-boswellic acid; ER: estrogen receptor; GC: gas chromatography; HPLC: high performance liquid chromatography; MS: mass spectrometry; PARP: poly (ADT-ribose) polymerase.

## Competing interests

CW and GY are affiliated with Young Living Essential Oils. The rest of authors declare that they have no competing interests.

## Authors' contributions

MS prepared *Boswellia sacra *essential oils. WW, AC, FGM, PTS, YTF, and HKL performed molecular and cell biology studies of cultured breast cells. MS, KMF, CW, GY, and HKL conceived the idea, designed the experiments, and interpreted the experimental and clinical results. All authors contributed to manuscript preparations and approved the final manuscript.

## Pre-publication history

The pre-publication history for this paper can be accessed here:

http://www.biomedcentral.com/1472-6882/11/129/prepub

## Supplementary Material

Additional file 1 **Table S1. Chemical composition of *Boswellia sacra *essential oil**. Chemical components of essential oil quantified by GC-MS. Click here for file
