# Supplementary material for: Boswellia sacra essential oil induces tumor cell-specific apoptosis and suppresses tumor aggressiveness in cultured human breast cancer cells
Source: BMC Complement Altern Med. 2011 Dec 15;11:129. doi: 10.1186/1472-6882-11-129 (PMC3258268; doi:10.1186/1472-6882-11-129)
Supplement: Additional file 1 — Table S1. Chemical composition of Boswellia sacra essential oil. Chemical components of essential oil quantified by GC-MS. [file 1472-6882-11-129-S1.DOC]

Table S1. Chemical composition of *Boswellia sacra* essential oil

|  | ***Boswellia sacra*** | Hydrodistillation temperature | |
| --- | --- | --- | --- |
| **Ret. Index** | **Component** | **78 ^o^C** | **100 ^o^C** |
| 755 | toluene | 0.10 | 0.06 |
| 922 | unidentified* | 1.03 | 0.80 |
| 929 | *alpha*-thujene | 0.98 | 0.93 |
| 940 | *alpha*-pinene | 65.49 | 59.40 |
| 953 | camphene + verbenene | 3.42 | 3.46 |
| 973 | sabinene | 2.12 | 3.63 |
| 979 | *beta*-pinene | 2.27 | 2.38 |
| 987 | myrcene | 7.46 | 5.36 |
| 994 | *ortho*-methyl anisole | 0.17 | 0.35 |
| 1004 | *alpha*-phellandrene | 1.20 | 1.30 |
| 1011 | *delta*-3-carene | 0.89 | 0.09 |
| 1018 | para-cymene | 1.24 | 1.23 |
| 1031 | limonene | 8.43 | 8.99 |
| 1039 | cis-*beta*-ocimene | 0.19 | 0.33 |
| 1055 | *gamma*-terpinene | 0.22 | 0.44 |
| 1079 | para-cymenene | 0.05 | 0.08 |
| 1085 | *alpha*-Terpinolene | 0.17 | 0.29 |
| 1101 | myrcenol | 0.06 | 0.11 |
| 1112 | *alpha*-campholene aldehyde | 0.30 | 0.70 |
| 1134 | trans-pinocarveol | 0.27 | 0.77 |
| 1138 | cis-verbenol | 0.22 | 0.46 |
| 1146 | unidentified** | 0.12 | 0.41 |
| 1149 | pinocamphone | 0.04 | 0.11 |
| 1153 | *alpha*-phellandren-8-ol | 0.22 | 0.80 |
| 1160 | borneol | 0.01 | 0.06 |
| 1166 | para-cymene-8-ol | 0.07 | 0.13 |
| 1171 | terpinene-4-ol | 0.14 | 0.50 |
| 1180 | *alpha*-terpineol + myrtenal | 0.12 | 0.40 |
| 1187 | myrtenol | 0.05 | 0.13 |
| 1191 | verbenone | 0.15 | 0.42 |
| 1203 | cis-carveol | 0.04 | 0.10 |
| 1243 | dimethyl ether orcinol | 0.06 | 0.10 |
| 1278 | bornyl acetate | 0.19 | 0.47 |
| 1339 | *alpha*-terpenyl acetate | 0.05 | 0.15 |
| 1391 | *alpha*-copaene | 0.06 | 0.13 |
| 1400 | *beta*-elemene | 0.41 | 0.94 |
| 1437 | trans-*beta*-caryophyllene | 0.28 | 0.62 |
| 1470 | *alpha*-humulene | 0.11 | 0.17 |
| 1477 | allo-aromadendrene | 0.02 | 0.06 |
| 1485 | *gamma*-muurolene | 0.05 | 0.10 |
| 1494 | germacrene D | 0.02 | 0.09 |
| 1501 | *beta*-selinene | 0.21 | 0.45 |
| 1509 | *alpha*-selinene | 0.12 | 0.24 |
| 1523 | *gamma*-cadinene | 0.04 | 0.07 |
| 1527 | *delta*-cadinene | 0.05 | 0.14 |
| 1593 | caryophyllene oxide | 0.02 | 0.05 |
|  | **Total** | **99.31** | **98.78** |

*unidentified m/z 93(100%), 41(54%), 67(39%), 69(36%), 91(33%), 77(29%), 79(26%), 39(25%), 108(15%), 53(14%), 55(12%), 121(9%)

**unidentified m/z 59(100%), 79(85%), 94(85%), 91(66%), 77(37%), 43(37%), 93(36%), 92(20%), 39(17%), 119(11%), 51(10%), 78(10%)
